# A wearable functional electrical stimulation device with a movable electrode for motor point tracking

**DOI:** 10.3389/fbioe.2025.1715337

**Published:** 2026-01-08

**Authors:** Yue Liu, Shin Ebihara, Masao Sugi, Hiroshi Yokoi, Yinlai Jiang

**Affiliations:** 1 Department of Mechanical and Intelligent Systems Engineering, The University of Electro-Communications, Tokyo, Japan; 2 Center for Neuroscience and Biomedical Engineering, The University of Electro-Communications, Tokyo, Japan

**Keywords:** functional electrical stimulation, motor point, muscle fatigue, rehabilitation, upper limb

## Abstract

Functional electrical stimulation is widely applied in the rehabilitation of individuals with cerebrovascular disease or spinal cord injury but is limited by rapid muscle fatigue. We present a novel stimulation strategy based on real-time motor point tracking using a wearable functional electrical stimulation device with a movable electrode. A crank–slider mechanism drives the electrode to follow the biceps brachii motor point trajectory according to elbow joint angle, aiming to optimize stimulation site and reduce fatigue. Seven healthy male participants compared this approach with time-shifted and joint angle–shifted stimulation. Muscle performance was evaluated by maximum voluntary contraction, change in elbow joint angle, and subjective comfort assessed on a visual analogue scale. Results showed that motor point tracking significantly reduced fatigue and improved comfort compared with conventional methods, supporting its potential to enhance functional electrical stimulation–based upper limb rehabilitation.

## 1 Introduction

Cerebrovascular disease and spinal cord injury (SCI), including stroke and other vascular lesions, remain among the leading causes of long-term disability worldwide (O’Flaherty and Ali, 2024). These conditions often result in motor, sensory, and cognitive impairments, with upper limb motor dysfunction being particularly disabling. Restoring fine motor functions such as grasping, reaching, and object manipulation remains a major challenge in neurorehabilitation due to its direct impact on activities of daily living, quality of life, and caregiver dependence (Momosaki et al., 2017; Hayward and Brauer, 2015).

Functional electrical stimulation (FES) is a widely adopted neuromodulation technique for motor recovery. By delivering electrical pulses to peripheral nerves or muscles, FES can evoke muscle contractions in individuals who lack voluntary motor control, thereby facilitating neural reactivation and promoting neuroplasticity (Chae et al., 2001; Marquez-Chin and Popovic, 2020; Carson and Buick, 2021). Clinical studies have demonstrated its potential to enable functional movements such as walking and reaching in patients with stroke and SCI (Meadmore et al., 2014).

Despite its utility, conventional FES systems face critical limitations, most notably the rapid onset of muscle fatigue (Buckmire et al., 2018; Schmoll et al., 2021). In natural voluntary movement, motor unit recruitment follows Henneman’s size principle: slow-twitch, fatigue-resistant fibers are recruited first, with fast-twitch fibers activated only when higher force is needed (Van Bolhuis and Holsheimer, 2001). In contrast, FES tends to preferentially activate fast-twitch fibers (Ibitoye et al., 2016). Furthermore, it induces synchronous activation of motor units, differing from the asynchronous pattern observed in voluntary contractions (Gregory and Bickel, 2005). These non-physiological recruitment patterns increase metabolic load and hasten fatigue, thereby limiting the duration and effectiveness of FES therapy. Although strategies such as adjusting stimulation frequency or duty cycle have been proposed (Thrasher et al., 2005; Vromans and Faghri, 2017), their effectiveness remains limited, particularly for multi-joint, multi-muscle tasks.

To mitigate muscle fatigue and improve stimulation efficacy, prior studies have focused on optimizing electrode placement by targeting the muscle’s motor point (MP)–the site of highest excitability and lowest activation threshold (Gregory and Coburn, 2018; Ibáñez et al., 2010; Narita et al., 2011). Anatomically, the MP is where the motor nerve enters the muscle belly; electrophysiologically, it is defined as the surface location that elicits maximal contraction with minimal stimulation. Accurate MP targeting can enhance contraction efficiency, reduce required stimulation intensity, and minimize discomfort–factors critical for sustained FES application.

In conventional muscle rehabilitation protocols, static isometric contractions are frequently employed, wherein the MP remains relatively fixed, allowing for straightforward localization and targeted stimulation (Deley et al., 2015; Jafari et al., 2025). However, in movement-driven rehabilitation tasks such as gait training, the MP position dynamically shifts in response to joint motion–Gonzalez et al. showed that the optimal stimulation site on the biceps brachii varies with elbow flexion angle: distal positions are more effective at smaller angles, while proximal sites are preferable at larger angles (Gonzalez et al., 2018). These findings emphasize the need to adjust electrode placement dynamically based on joint kinematics, rather than relying on predefined static positions–particularly for muscles like the biceps and triceps, which exhibit significant MP displacement during movement.

**FIGURE 1:**
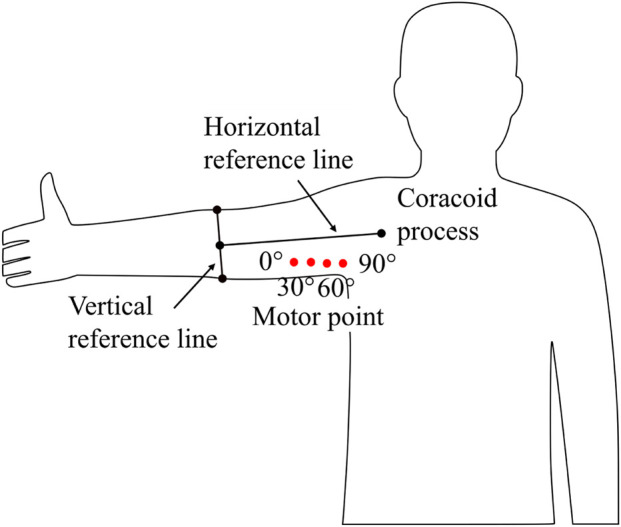
The variation trend of the MP of the right upper arm biceps brachii with respect to elbow flexion angle. To quantify the coordinates of the MP position, the vertical reference line is defined along the circumference line of the elbow joint, while the horizontal reference line is defined as the line passing through the coracoid process and perpendicular to the vertical reference line.

Building on this, we quantitatively mapped the MP position of the biceps brachii and expressed it as a pair of spatial coordinates. A schematic representation of this mapping is shown in Figure 1.

These insights suggest that predetermined stimulation position is insufficient for dynamic upper-limb tasks. Instead, real-time MP tracking systems are required to maintain effective stimulation across the full range of motion, offering improved selectivity, reduced fatigue, and better clinical outcomes.

To address the limitations of predetermined stimulation position in FES, Ichikawa et al. proposed motor point tracking stimulation (MPTS) with multiple stimulation electrodes, a method that dynamically adjusts the stimulation site in real time according to joint motion, keeping the electrode aligned with the muscle’s MP. This approach improves stimulation efficiency, reduces muscle fatigue, and enhances FES performance during dynamic movements (Ichikawa et al., 2021). The MPTS includes two implementation strategies: time-based shifting stimulation (TSS) and joint-angle-based shifting stimulation (JASS). TSS changes the stimulation position according to a predefined time sequence, assuming a fixed relationship between time and joint motion. JASS, in contrast, switches the stimulation site based on real-time joint angle data, allowing more accurate targeting of the MP during movement. Ichikawa demonstrated that the three-channel JASS protocol is more effective than TSS in mitigating muscle fatigue. Furthermore, increasing the number of stimulation channels and employing smaller electrodes can enhance this effect. However, the reduced electrode size leads to higher current density, which may result in discomfort and pain during stimulation (Crochetiere et al., 1967).

To address the limitations of conventional MPTS systems with static electrodes–particularly their reliance on bulky actuators and small-sized electrodes–we propose a novel strategy termed movable electrode tracking stimulation (METS). This approach features a single surface electrode that passively tracks the dynamic displacement of the MP along the skin in response to joint movement, facilitated by a compact crank-slider transmission coupled to elbow flexion. The proposed design circumvents the discomfort associated with high current densities and eliminates the weight and power constraints inherent in motorized systems, thereby enhancing both comfort and practicality for real-world rehabilitation applications.

To improve tracking precision, we introduce a nonlinear regression model that characterizes MP displacement as a function of elbow joint angle, enabling more accurate electrode positioning than conventional TSS or JASS methods.

We implemented this concept as a lightweight, wearable METS device optimized for upper-limb applications. Its modular and flexible design allows easy adaptation to a range of user anatomies, enhancing both comfort and usability. To the best of our knowledge, this is the first METS implementation that combines passive mechanical tracking with nonlinear trajectory modeling in a compact wearable form. These contributions mark a significant step toward practical, fatigue-reducing, and user-adaptive FES systems for real-world rehabilitation.

## 2 Materials and methods

### 2.1 METS device

According to the average data of people aged 20 to 60 (Chow et al., 2020), when the elbow joint is bent from 0° to 90°, the MP of the biceps moves about 32 mm. Since this study uses the surface electrode method for FES, it is necessary to always maintain good contact between the electrode and the skin and meet the requirements of the device that can be used in various environments. The improvement goals are: (i) to reduce the number and size of motors used in the device, thereby reducing the overall weight of the device and making it easier to wear. (ii) maintain good contact between the electrodes and the MP when the user moves the joint.

**FIGURE 2:**
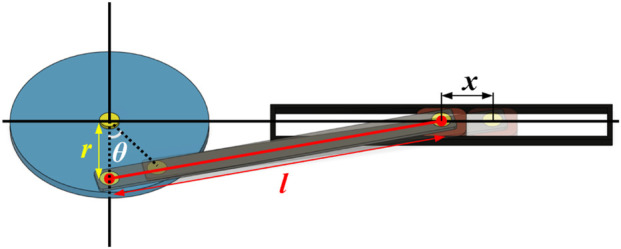
The slider-crank structure used in the METS device, r represents the pulley radius equivalent to the crank length, l represents the connecting rod length, and θ represents the central angle of the equivalent crank rotation.

The crank slider transmission structure shown in Figure 2 is a structure that satisfies both conditions. The pulley is rigidly connected to the forearm handle. When the elbow joint is bent, the handle fixed on the forearm drives the pulley to rotate. The crank slider mechanism connected to the pulley converts the rotation of the elbow joint into linear motion, thereby driving the electrode to slide on the skin. The displacement of the slider is determined by the length of the active handle and the driven handle, and its calculation formula is shown in Equation 1.

$$x=r\left\{\sin\theta +\sqrt{{\left(\frac{l}{r}\right)}^{2}-\cos^{2}\theta }-\sqrt{{\left(\frac{l}{r}\right)}^{2}-1}\right\}$$ (1)

where x represents the displacement of the slider–corresponding directly to the displacement of the electrode–and θ represents the angle of the elbow.

In the experiment, in order to determine the relationship between the MP position and the elbow joint angle, subjects were asked to move their elbows from 0° to 90° in 15° increments, and the MP position at each angle was measured using a motor point pen. To ensure measurement reliability, five repeated measurements were performed at every angle, with subjects relaxing their upper limb for approximately 20 s between measurements. The coordinate of each MP point was recorded relative to the vertical reference frame shown in Figure 1, and the averaged value was used for subsequent analysis. The MP position at 0° was defined as the zero-displacement reference, and the change in MP position with elbow angle was plotted. The least squares method was then applied to approximate this trajectory with a quadratic polynomial, as shown in Equation 2.

$$L\left(\theta \right)=a_{0}+a_{1}\theta +a_{2}\theta^{2}$$ (2)

where $L\left(\theta \right)$ represents the displacement of the MP and θ represents the angle of the elbow.

**FIGURE 3:**
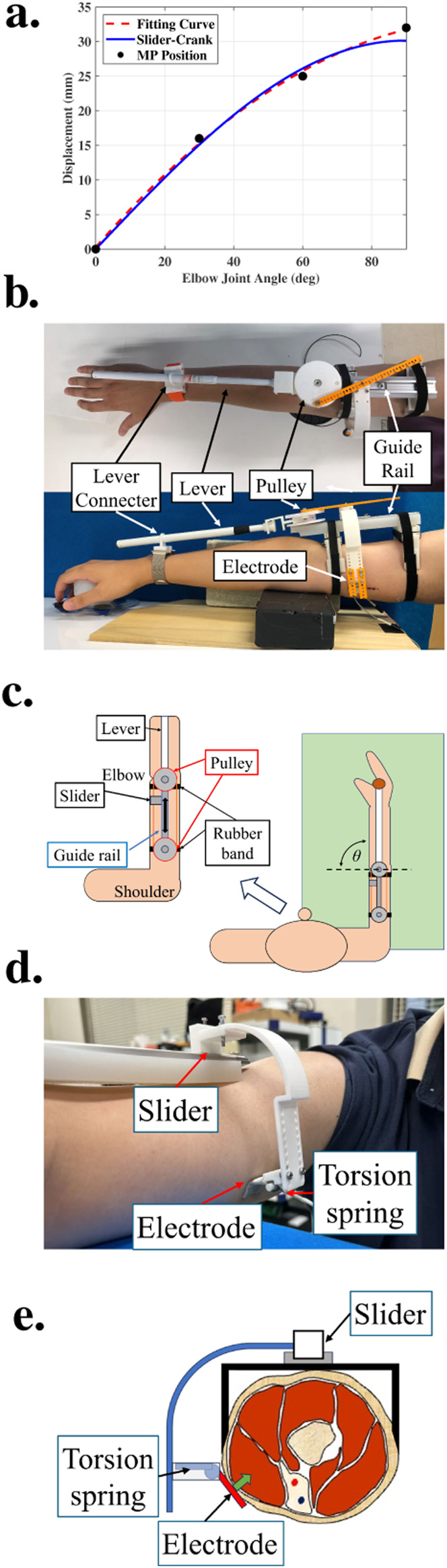
**(a)** Comparison between the actual MP position and the fitted curve derived from the MP trajectory, together with the corresponding angle–displacement relationship of the slider–crank mechanism. **(b)** External appearance of the METS device when worn. **(c)** Schematic diagram illustrating the working principle of the METS device. **(d)** Electrode fixation structure located near the biceps MP. **(e)** Schematic representation of the electrode fixation mechanism.

The displacement matching between the actual MP trajectory and the slider–crank mechanism is shown in Figure 3a. Figures 3b,c shows the prototype of the METS device. The pulley has connection points every 0.5 mm in the radius range of 25–28 mm, and connection points are set every 5 mm on the driven handle. The value of $\frac{l}{r}$ can be adjusted by changing the connection point. The values of r and l required for each subject were determined by solving the optimization problem shown in Equation 3. Sample data from one subject shown in Figure 3a, indicating that the displacement generated by the crank–slider mechanism closely matched the subject’s actual MP trajectory, with a maximum error of only 1.8 mm at a joint angle of 90°. In practice, since elbow flexion stops at approximately 90° and FES terminates simultaneously, the electrode–MP displacement error during stimulation remained within this range.

The stimulating electrode used in the experiment was a circular dry electrode with a radius of 7 mm, which was sufficient to cover the MP within the observed error range. Its surface was treated to allow smooth sliding on the skin when conductive gel was applied. The gel was used primarily to reduce sliding friction between the electrode and the skin, and it remained sufficiently moist throughout the duration of the experimental sessions without affecting stimulation performance. In combination with the torsion spring structure shown in Figures 3d,e, appropriate pressure was applied to ensure stable electrical contact between the electrode and the skin during the entire experiment.

$$\underset{\frac{l}{r}}{\mathrm{arg min}}\int_{0}^{\frac{\pi }{2}}\left|L\left(\theta \right)-x\right|\;d\theta$$ (3)

### 2.2 Subjects

The subjects were 7 healthy males aged 24 ± 3 years (mean ± standard deviation). The study was approved by the Ethics Committee of the University of Electro-Communications [No.10006(7)] and was carried out according to the principles of the Declaration of Helsinki. Furthermore, subjects were informed of the benefits and risks of the experiment, the procedures, the FES device to be used, and the use of experimental equipment, and written informed consent was obtained prior to the experiment.

### 2.3 Experimental protocol

To compare the effects of the proposed method with TSS and three-channel (3-Ch) JASS on the subjects, each subject underwent three stimulation experiments. As a controlled variable, all three experiments followed the same stimulation protocol: a cycle consisting of 2 s of stimulation followed by 3 s of relaxation. The 2 s stimulation period was sufficient for all subjects to achieve elbow flexion and did not cause any discomfort within a single cycle. During the stimulation phase, contraction of the biceps brachii induced elbow flexion, while in the relaxation phase the muscle was released and the arm naturally returned to the initial position (0° between upper arm and forearm). The experiment was carried out for 180 cycles (15 min), sufficient to reach the average fatigue threshold under electrical stimulation (Zheng and Hu, 2018; Sayenko et al., 2014; Rongsawad and Ratanapinunchai, 2018).

**FIGURE 4:**
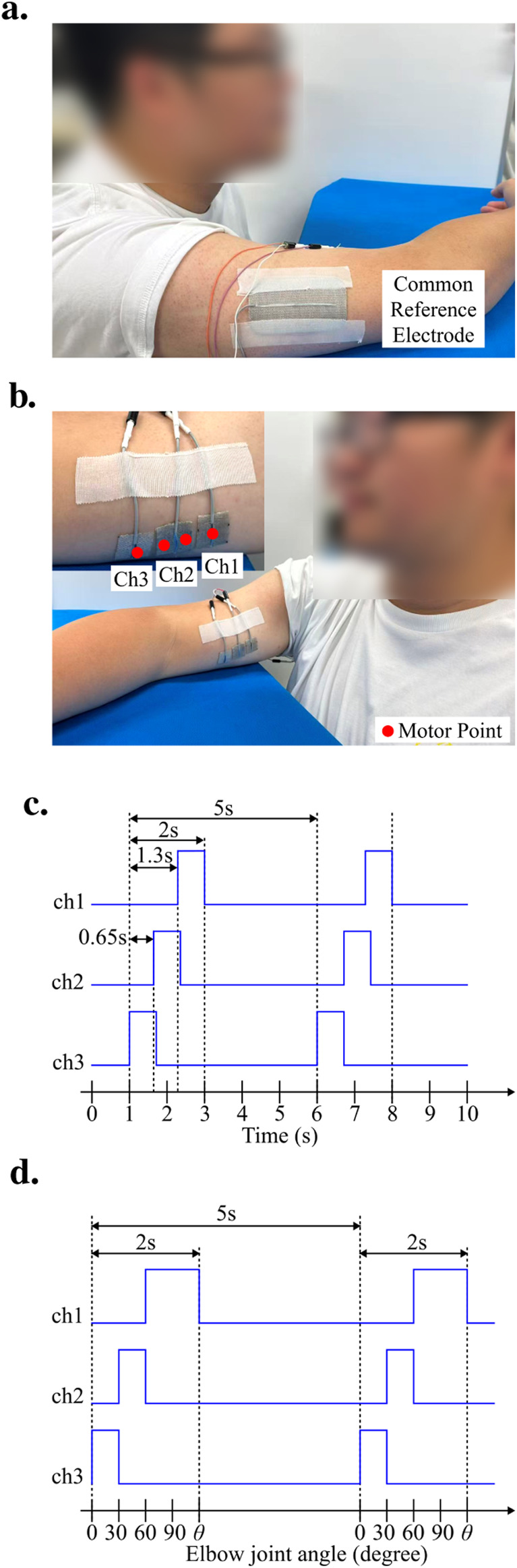
**(a)** Placement of the common reference electrode on the triceps side of the subject. **(b)** Arrangement of the three stimulation channels on the biceps side, together with their relative positions to the MP at elbow joint angles of 0°, 30°, 60°, and 90°. **(c)** Stimulation pattern in the TSS experiments, where each channel was activated for 0.7 s with an inter-channel delay of 0.65 s to ensure smooth transitions between channels. **(d)** Stimulation pattern in the 3-Ch JASS experiments, with the horizontal axis indicating elbow joint angle, which differs from that in the TSS pattern.

The experimental configurations of TSS and JASS are shown in Figure 4. As illustrated in Figures 4a,b, both TSS and JASS employed the same three stimulation electrodes, referred to as channel 1 (Ch1), channel 2 (Ch2), and channel 3 (Ch3) placed along the biceps brachii. Specifically, Ch3 was positioned to cover the motor point (MP) corresponding to the elbow joint angle of 0°, Ch2 was placed over the MPs associated with elbow flexion angles of 30° and 60°, and Ch1 was located at the MP for 90°.

By adopting different electrode activation strategies, stimulation of distinct MPs could be achieved. The difference between the two methods lies in the activation scheme: in TSS, each stimulation phase of a cycle was divided into three equal segments, and the three electrodes were sequentially activated in time order to achieve a tracking-like effect. In contrast, JASS employed a position sensor to calculate the elbow joint angle in real time, and the electrode was activated according to the angle range: Ch3 for 0° to 30°, Ch2 for 30° to 60°, and Ch1 for 60° to. 90°.

**FIGURE 5:**
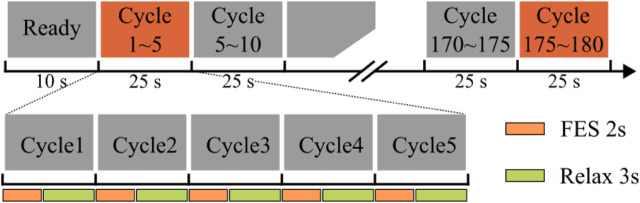
Experimental protocol. The experiment consisted of 180 consecutive stimulation cycles. Each cycle lasted 5 s, comprising 2 s of electrical stimulation followed by 3 s of rest. Stimulation induced elbow flexion, and during rest the elbow joint returned to its initial position.

The experimental procedure is illustrated in Figure 5. Each subject underwent the three stimulation techniques in a randomized order, with an interval of at least 48 h between sessions to ensure full recovery and avoid confounding fatigue. Subjects were also instructed to refrain from high-intensity exercise before and during the experiment.

To evaluate whether electrode movement during METS operation affects the electrical stability of the skin–electrode interface, we measured the skin–electrode impedance continuously throughout the stimulation period. This analysis aimed to determine whether sliding motion would introduce additional impedance variability that might influence current delivery or stimulation performance.

### 2.4 Stimulation parameters

The stimulation device was a self-developed constant-voltage, frequency-modulated stimulator that allowed configuration of carrier and burst waveform parameters. The detailed stimulation parameters are listed in Table 1. Constant-voltage stimulation was selected because it is highly suitable for wearable and battery-powered FES devices. Unlike constant-current systems, a voltage-based stimulator does not require continuous adjustment to compensate for impedance fluctuations, avoiding the need for high-power boost circuits and reducing overall electrical losses. This results in higher energy efficiency and longer battery life, which are essential for portable applications. In addition, the simpler circuit architecture of constant-voltage stimulation enables a more compact and lightweight design, facilitating integration into wearable hardware.

**TABLE 1:** Parameters of the three stimulation methods.

Parameters	TSS	3-Ch JASS	METS
Carrier frequency	2000 Hz	2000 Hz	2000 Hz
Carrier duty cycle	50%	50%	50%
Burst frequency	200 Hz	200 Hz	200 Hz
Burst duty cycle	50%	50%	50%

The stimulation voltage was determined individually for each subject prior to the fatigue experiment (Sun et al., 2024; Malešević et al., 2012). With electrodes fixed in place, the voltage was increased from 0 V in steps of 0.2 V, each maintained for 5 s, until either noticeable discomfort was reported or visible biceps brachii contraction was observed. The voltage at that point was recorded, then reduced by 0.2 V to define the stimulation level for the formal experiment. This ensured elbow flexion of at least 90° without causing pain. The same voltage value was used across the three techniques for each subject. The final stimulation voltages for all subjects are shown in Table 2.

**TABLE 2:** Age and stimulation voltage of the seven subjects.

Subject	Age	Voltage
1	21	7.6 V
2	22	5.8 V
3	27	7.0 V
4	26	6.6 V
5	24	5.6 V
6	24	6.0 V
7	24	6.6 V

### 2.5 Muscle fatigue evaluation

To evaluate muscle fatigue based on muscle strength, maximum voluntary contraction (MVC) was measured before and after each fatigue experiment. During the measurement, a dynamometer was fixed at an appropriate position on the workbench so that the pulling force exerted by the subject was aligned perpendicularly to the force-sensing element. Subjects were instructed to maintain the elbow joint at a constant angle of 90° and to wear a wrist brace with a metal liner, ensuring that only the upper arm contributed to exerting maximal force during isometric contraction. Each MVC measurement was repeated three times, with each contraction sustained for 3 s and followed by a 1 min rest interval. The final MVC value was calculated as the average of the three trials (Todd et al., 2003; Wang et al., 2023; Zellers et al., 2019). To quantify the degree of muscle fatigue, the relative change in MVC was calculated using Equation 4.

$$F_{i,j}=\frac{a_{i,j}-b_{i,j}}{a_{i,j}}\times 100$$ (4)

where $F_{i,j}$ represents the percentage decrease in MVC for subject j during the i th muscle fatigue trial. $a_{i,j}$ and $b_{i,j}$ are the pre- and post-fatigue MVC values, respectively.

To evaluate muscle fatigue based on joint movement characteristics, an inertial motion tracking sensor (Viper8, The Micro Sensor 1.8, Polhemus) was used to continuously record the elbow joint angle in real time during the experiment, with a sampling frequency of 240 Hz. Cycles 1–5 and 175–180 were selected as representative data before and after stimulation, respectively. This selection captures the dynamic range at the beginning and end of the stimulation session to reflect changes in motor performance over time. The change rate of elbow joint angle was calculated using Equation 5.

$$G_{i,j}=\frac{\sum_{k=1}^{5}\left(r_{i,j,k}\right)-\sum_{k=176}^{180}\left(r_{i,j,k}\right)}{\sum_{k=1}^{5}\left(r_{i,j,k}\right)}\times 100$$ (5)

where $G_{i,j}$ represents the rate of change of elbow joint angle of j th subject during i times muscle fatigue stimulation. r represents the maximum elbow joint angle of each cycle and k represents the number of cycles.

To comprehensively evaluate participants’ subjective experiences during the experiment, a Visual Analog Scale (VAS) was employed to assess discomfort, fatigue, and muscle responses associated with the device. The VAS is widely used in both clinical and experimental settings due to its simplicity, sensitivity, and ability to capture nuanced changes in subjective perception (Tubach et al., 2012; Jeon and Griffin, 2018). In this study, participants completed VAS evaluations immediately after wearing the device and again after each stimulation session. The specific evaluation items are listed in Table 3.

**TABLE 3:** VAS evaluation items.

Status	VAS items
After wearing	(a) Mental discomfort
	(b) Physical discomfort
	(c) Time spent wearing
	(d) Mental discomfort
	(e) Physical discomfort
After stimulation	(f) The presence of the device
	(g) Pain sensation
	(h) Feeling of fatigue
	(i) Muscle response to stimulation

To ensure consistency and reliability of the assessments, all VAS procedures followed a standardized protocol (Heller et al., 2016; Byrom et al., 2022; Delgado et al., 2018). Each item was presented as a 100 mm horizontal line anchored with descriptive labels at each end (e.g., “no discomfort” to “maximum imaginable discomfort”). Participants were instructed to place a mark on the line that best represented the intensity of their experience. The distance from the left end to the mark was measured in millimeters, yielding a score between 0 and 100. All evaluations were conducted in a quiet environment under researcher supervision to minimize bias and ensure accuracy.

## 3 Results

### 3.1 Comparison of impedance characteristics

**FIGURE 6:**
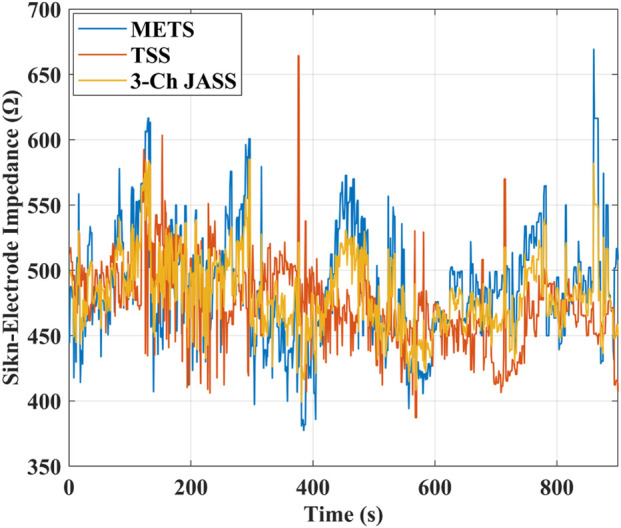
Skin–electrode impedance measured during stimulation for the sliding electrode used in METS and the fixed electrodes used in TSS and 3-Ch JASS. Three impedance curves exhibit only small fluctuations throughout the stimulation period, with no abrupt changes or monotonic trends. The variability observed in the sliding electrode is comparable to that of the fixed electrodes, indicating that electrode movement does not introduce additional instability at the electrode–skin interface.

Skin–electrode impedance during stimulation is shown in Figure 6 for both the sliding electrode used in METS and the fixed electrodes used in TSS and 3-Ch JASS. Across the entire stimulation period, the impedance curves exhibited only small fluctuations, with no abrupt changes or monotonic trends. The variability observed in the sliding electrode was comparable to that of the fixed electrodes, indicating that electrode movement did not introduce additional instability at the electrode–skin interface.

The magnitude of these fluctuations remained within the typical physiological impedance range reported in the literature. Given the constant-voltage stimulation scheme used in this study, such mild impedance variations did not produce noticeable changes in stimulation intensity or muscle contraction. Therefore, we concluded that the electrode–skin impedance remained sufficiently stable throughout the experiment and was unlikely to be a major factor affecting the stimulation outcome or fatigue-based comparisons among the three methods.

### 3.2 Changes in MVC under different stimulation modes

**FIGURE 7:**
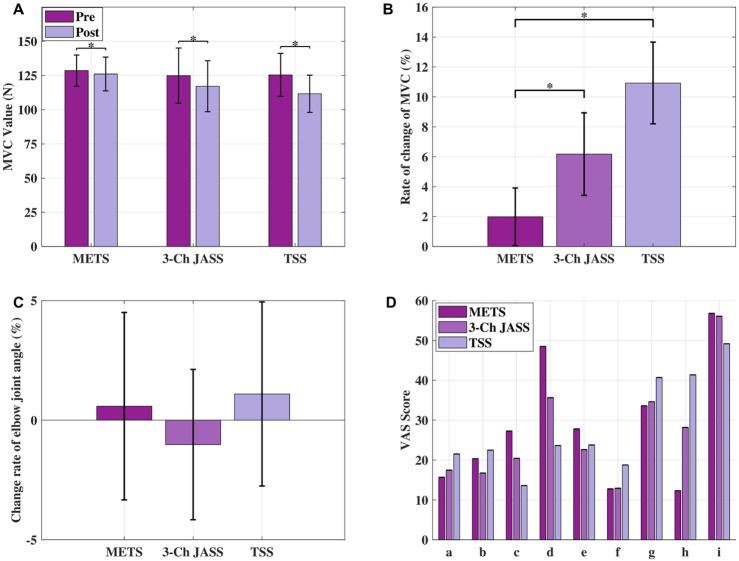
Changes in muscle fatigue indicators and subjective evaluations under three stimulation modes. **(A)** MVC of the biceps brachii. **(B)** Rate of change in MVC, reflecting muscle fatigue level. **(C)** Change rate of elbow joint angle per cycle at the beginning and end of the experiment.**(D)** VAS results from participants; specific evaluation items are listed in Table 2.

The MVC average values of METS, TSS, and 3-Ch JASS are shown in Figure 7a. The error bars indicate the standard deviation. Statistical analyses were performed using non-parametric methods because the Shapiro–Wilk test indicated that the MVC data did not follow a normal distribution (p<0.05) . The Shapiro–Wilk test is commonly used to assess whether sample data follow a normal distribution, and violation of normality assumptions suggests the need for non-parametric procedures.

To evaluate the effect of each stimulation strategy on muscle fatigue before and after stimulation, the Wilcoxon signed-rank test was applied. This test is appropriate for dependent samples with non-normal distributions and assesses systematic differences between paired measurements, such as pre- and post-stimulation MVC values.

For comparing the MVC change rates among the three stimulation methods, Steel’s method was used with METS as the reference group. Steel’s method is a non-parametric multiple comparison procedure that allows several treatments to be compared against a single control without assuming normality. These analyses were employed to identify statistically significant differences in fatigue-related outcomes across stimulation strategies. In Figure 7a, * indicates p<0.05 .

Figure 7b shows the average change rate of MVC, with error bars representing standard deviations. Consistent with the above analysis, Steel’s method was used to evaluate differences between METS and the two comparison conditions (TSS and 3-Ch JASS). As indicated in the figure, * denotes statistical significance at the 5% level.

### 3.3 Changes in elbow flexion angle under different stimulation modes

The change rates of joint angle for METS, TSS, and 3-Ch JASS are presented in Figure 7c, with error bars representing standard deviations. It can be observed that an abnormal negative change rate appeared in the 3-Ch JASS condition. We attribute this to the relatively large inter-subject variability and the small absolute values of joint angle changes, where slight incidental movements made by the subjects during the experiment may have affected the results. Nevertheless, no significant differences were observed among the three methods, and the change rates remained within approximately 1%, indicating that 20 min of stimulation did not result in a notable alteration in joint angle.

Previous studies have shown that excessive muscle fatigue can lead to increased variability or instability in joint kinematics, including significant changes in joint angles during repetitive or load-bearing tasks Gates and Dingwell (2011). In contrast, the stable joint angle observed in our experiment suggests that no excessive fatigue occurred during stimulation.

In this study, the stimulation voltage was uniformly set to the highest level tolerable without pain, and the stimulation intensity was standardized as the amount required to induce 90-degree elbow flexion. Therefore, from the perspective of elbow joint movement, the stimulation conditions applied in our protocol did not induce excessive fatigue.

This confirms the validity of the experimental design and supports the interpretation that the observed decline in MVC truly reflects the muscle fatigue induced by the respective stimulation methods, rather than being confounded by excessive fatigue-related motor dysfunction.

### 3.4 Subjective evaluation by VAS survey

The VAS results shown in Figure 7d suggest that METS induced greater mental discomfort compared with TSS and 3-Ch JASS. According to feedback from the subjects, this discomfort primarily arose from the requirement to wear a visible mechanical structure, whereas the other two methods only involved attaching surface electrodes. This highlights the importance of device design in influencing user acceptance. Nevertheless, METS demonstrated a distinct advantage in reducing perceived muscle fatigue, supporting the effectiveness of the proposed strategy in mitigating fatigue during FES. The slightly longer wearing time of METS, due to the need for subject-specific adjustments of the pulley radius and driven shaft length, reflects a trade-off between functionality and usability. Importantly, no significant differences were observed among the three methods in other evaluation items, suggesting that METS does not introduce additional burden beyond the noted issues of psychological discomfort and preparation time.

## 4 Discussion

For the three different stimulation methods, MVC showed a significant decrease after the experiment, indicating that the stimulation protocols induced muscle fatigue. Among the methods, METS demonstrated the smallest MVC change rate, significantly lower than those of 3-Ch JASS and TSS. This suggests that METS has a clear advantage in reducing muscle fatigue compared with traditional multi-channel strategies. In addition, the order of fatigue magnitude–TSS, followed by 3-Ch JASS, then METS–was consistent with the findings of Ichikawa et al., further supporting the effectiveness of joint-angle-based motor point tracking. While MVC was selected as a practical and widely used indicator for comparing fatigue among different stimulation strategies, we acknowledge that relying solely on MVC provides a limited assessment of muscle fatigue. In future work, we plan to incorporate additional quantitative measures–such as EMG amplitude and spectral characteristics, M-wave responses, and contraction-based indicators–within an online EMG-FES evaluation framework to achieve a more comprehensive assessment of fatigue and further clarify the differences among METS, TSS, and 3-Ch JASS.

In analyzing the change rate of joint angle during the experiment, no significant differences were found among the three methods, indicating that 20 min of stimulation did not noticeably alter joint angle behavior. Because the stimulation voltage and intensity were calibrated to achieve 90° elbow flexion without pain, it can be inferred that the stimulation level used in this study did not cause substantial joint-level fatigue.

We also acknowledge that the current prototype may impose constraints on upper-limb movement freedom due to its mechanical structure. The design was developed with the target population in mind–particularly individuals with spinal cord injury or vascular lesions, who often present limited voluntary upper-limb mobility such as reduced pronation and supination. For this reason, the potential influence of the device on movement freedom was not evaluated in healthy subjects. Nonetheless, considerations regarding movement restriction are important for future applications. Future iterations of the METS system will incorporate structural optimization to reduce device bulk and minimize interference with natural joint kinematics.

While conductive gel was effective for reducing sliding friction during the experimental session, we acknowledge that its long-term stability may become a limiting factor. For extended or daily use scenarios, gel drying could degrade user experience and reduce the reliability of sustained stimulation. To address this, the METS device is designed to be compatible with dry electrodes. Dry operation can be facilitated by applying a low-friction surface coating or incorporating a hydrophilic polymer membrane at the electrode–skin interface to maintain stable contact during sliding. These improvements will be explored in future iterations of the device to enhance practicality in long-duration or clinically deployed settings.

The VAS results also highlight potential directions for improving the current prototype. The device could be optimized in material selection and wearing comfort, for example, by adopting flexible structures or integrating the torsion spring with the sliding guide to reduce discomfort and preparation time. Furthermore, extending the applicability of METS to other joints, such as the wrist or lower limbs, will require tailored mechanical adaptations to ensure usability across different anatomical regions. For future clinical use–especially among individuals with stroke or spinal cord injury–additional challenges such as spasticity, increased subcutaneous fat, altered muscle tone, and irregular movement trajectories must be considered. These aspects will guide the refinement of both mechanical design and stimulation control strategies in our subsequent research.

## 5 Conclusion

This study proposed and validated a novel wearable FES device termed as METS, which is incorporating the MPTS strategy based on a mechanical slider-crank structure. Unlike traditional methods such as TSS and multi-channels JASS, the METS employs a movable electrode that dynamically follows the MP during elbow flexion.

Through experiments involving seven healthy male subjects, the device was evaluated based on both objective measures (MVC, joint angle change) and subjective feedback (VAS scale). Results demonstrated that the proposed METS device significantly mitigated muscle fatigue without compromising stimulation precision or range of motion. Although the device introduced slightly more discomfort due to its added weight and setup complexity, it showed superior performance in fatigue reduction and muscle responsiveness.

Notably, the implementation of a compact, modular, and anatomically adaptable mechanism enables personalized MP tracking for users of different body sizes. This work represents the first successful integration of mechanical motion conversion with MP-aligned stimulation in a wearable format, offering a promising direction for fatigue-aware, user-friendly FES solutions in neurorehabilitation. Future studies may extend this approach to clinical populations and optimize real-time control algorithms for broader functional movement tasks.

## Data Availability

The raw data supporting the conclusions of this article will be made available by the authors, without undue reservation.
